# South African palliative care provider perspectives on emergency medical services in palliative situations

**DOI:** 10.1016/j.afjem.2024.08.007

**Published:** 2024-08-30

**Authors:** Caleb Hanson Gage, Liz Gwyther, Willem Stassen

**Affiliations:** a Division of Emergency Medicine, Faculty of Health Sciences, University of Cape Town, Cape Town, South Africa; b Division of Interdisciplinary Palliative Care and Medicine, Faculty of Health Sciences University of Cape Town, Cape Town, South Africa

**Keywords:** EMS, Paramedic, Palliative care, End-of-life, Homecare

## Abstract

**Introduction:**

Due to the frequent intersection of Emergency Medical Services (EMS) with palliative situations and the increasing global need for palliative care, there has been increased recognition of the need for palliative care integration with EMS. However, EMS and palliative care systems remain segregated in many Low-to-Middle Income Country contexts, as in South Africa (SA). The aim of this study was to gather perspectives of palliative care providers in SA concerning EMS in palliative situations.

**Methods:**

A qualitative design employing individual semi-structured interviews was implemented. Ten interviews with experienced doctors and nurses holding post-graduate palliative medicine qualifications were conducted. Verbatim transcriptions of interviews were subjected to content analysis with an inductive-dominant approach to develop codes and categories.

**Results:**

Four categories were developed: (1) Disposition towards EMS, (2) Perceived EMS challenges, (3) Positive EMS impact across patients’ palliative care journeys and (4) Methods of EMS and palliative care system integration. Participants maintained an overall positive view of EMS and palliative care integration, noting the beneficial impact of EMS and suggesting various methods of integration, while also highlighting challenges and concerns.

**Conclusion:**

EMS and palliative care integration would be mutually beneficial to both systems while benefiting patient well-being and the broader healthcare system. Potentially low-cost, high-impact interventions suggested by participants, such as palliative care cards for patients and enhancing EMS and palliative care system communication, represent efficacious and judicious use of limited resources within the SA context. Pilot studies investigating these suggestions should be conducted.

**African relevance**

• Africa contains disproportionately high palliative care needs when compared with the rest of the world due to large disease burdens coupled with rapidly increasing and ageing populations resulting in high prevalence of life-limiting illnesses.
• Despite these needs, access to palliative care within Africa remains inadequate as palliative care systems are underdeveloped and resources are constrained.
• To meet the high demand for palliative care on the continent, palliative care integration with other disciplines, such as Emergency Medical Services (EMS), has been recommended.
• As EMS systems continue to develop within Africa it is imperative that integration with palliative care begin.

Alt-text: Unlabelled box

## Introduction

Palliative care is *‘an approach that improves the quality of life of patients and their families facing the problems associated with life-threatening illness, through the prevention and relief of suffering by means of early identification and impeccable assessment and treatment of pain and other problems, physical, psychosocial and spiritual’* [1]. Palliative care is integrative in nature, incorporating a broad spectrum of healthcare disciplines, including emergency medicine, to provide holistic patient care [1,2]. Recently, there has been increased recognition of the need for palliative care integration in out-of-hospital emergency medical services (EMS) [[3], [4], [5], [6]]. This is due to the frequent intersection of EMS with palliative situations [7] and the increasing global need for palliative care [8]. For the purposes of this study, ‘palliative situation’ is defined as any event involving the care of a patient with palliative needs, including emergency, non-emergency, and end-of-life situations.

Progress has been made with EMS and palliative care integration in various high-income countries (HICs), such as Australia [5] and Canada [9]. Part of the approach in these contexts has been gaining the perspectives of EMS and palliative care providers, patients, and family members on EMS in palliative situations [10,11]. Despite raising some concerns, such as a lack of EMS provider palliative care training, these role-players have generally viewed EMS involvement in palliative situations positively [11]. However, palliative care provider perspectives are relatively scarce and further research, particularly in low-to-middle income countries (LMICs), has been recommended [4].

Within the LMIC context of South Africa (SA), EMS and palliative care systems remain segregated [12]. There is little research on the topic in SA, and within LMICs more generally, despite a greater need for palliative care when compared with HICs due to disproportionately high burdens of disease and life-limiting complications thereof [4]. SA literature which does exist has demonstrated frequent intersection between EMS and palliative situations [12] and has gained EMS provider perspectives on palliative care [13]. However, palliative care provider perspectives concerning EMS are absent in the country. To assist in filling this knowledge gap, our study aimed to gather perspectives of palliative care providers in SA concerning EMS in palliative situations.

## Methods

### Design

A qualitative design employing individual semi-structured interviews was implemented. Husserlian descriptive phenomenology, which seeks to describe the lived experience of participants, provided the underlying theoretical framework [14].

### Setting

SA maintains two healthcare sectors: private and state [15]. State healthcare is provided by the government to all citizens while private healthcare is accessible exclusively to those with adequate financial means or healthcare insurance. EMS are, likewise, divided into these sectors. Out-of-hospital emergency care is provided using a paramedic-led rather than physician-led model [13]. Formal higher education (HE) training is required to register as an EMS provider, however, this requirement is recent, and many providers with vocational training remain registered and practicing [16]. HE courses range from one (assistant) to four years (practitioner) in duration.

Most SA palliative care is provided by non-governmental organizations (NGOs) which provide hospice services [17]. These NGOs are registered as charities and are reliant on donations and volunteers including doctors, nurses and social workers. As of 2018, 150 hospices and 8 hospital-based palliative care services exist in SA [18]. However, inequalities exist in the distribution of these facilities and many areas of the country lack access to palliative care.

### Data collection

An interview schedule was jointly developed by the authors based on contextualisation of current literature and previous work by Gage et al. [4,13]. The interview schedule (Supplementary Material 1) contained six questions referencing career background, utility, barriers, concerns, and feasibility *vis-à-vis* EMS use for palliative situations in SA. Pre-determined prompts and probes were included for each question to enhance in-depth exploration of participant perspectives and limit the introduction of interviewer biases.

Interviews were performed virtually by CG using Microsoft Teams™, with cameras, audio-recording, and transcription enabled, at an agreed upon time. A pilot interview was performed prior to data collection by CG and LG. Data from this interview has been included in the final report as LG met inclusion criteria and content from this pilot aligned with the other interviews. Interviews were conducted one-on-one with no third parties present.

Palliative care providers were contacted *via* email circulation through palliative and hospice databases: The Association of Palliative Care Practitioners of South Africa (PALPRAC), Association of Palliative Care Centres (APCC), University of Cape Town (UCT) Division of Interdisciplinary Palliative Care and Medicine. PALPRAC is a non-profit organization made up of SA doctors trained in palliative medicine [2]. The APCC is a national association of hospices with members from all nine SA provinces [19]. The UCT Division of Interdisciplinary Palliative Care and Medicine is the section of the university dedicated to palliative care education [20]. A brief description of the study was presented and interested providers meeting inclusion criteria signed a consent form.

Inclusion criteria were palliative care providers with post-graduate palliative qualifications (e.g. Post-Graduate Diploma) with a minimum of two years palliative care experience in SA. Exclusion criteria were palliative care providers other than nurses and medical doctors.

Eight interviews, including the pilot, were held from April to October 2023 meeting recommendations for phenomenological studies [21]. After analysis, two further interviews were performed in March 2024 to confirm saturation. Four participants knew of the interviewer from previous collaborations and all participants were aware the study formed part of the interviewer's post-graduate studies. No participants refused or dropped out of the study. No repeat interviews were conducted.

### Analysis

**Fig. 1:**
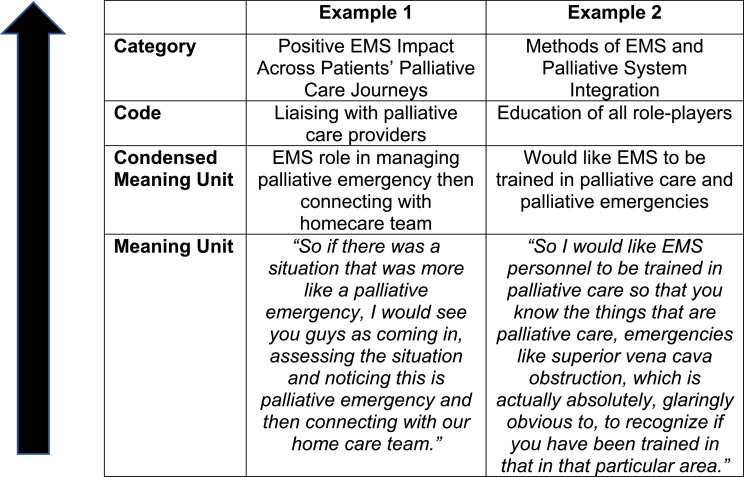
Coding tree.

Content analysis was performed using the framework of Erlignsson and Brysiewicz with an inductive dominant approach [22]. Transcriptions were read repeatedly for familiarisation after which initial coding was performed. Meaning units were captured, condensed, coded and categorized using NVivo version 14 (Fig. 1). Categories were then reviewed, refined, named, defined and written in the final report. These steps were performed alongside data collection as field notes were recorded during interviews.

CG independently analysed the data. Researcher triangulation was then performed between all authors where codes and categories were discussed and finalised. Data saturation, as detailed by Saunders et al., [23] was confirmed after ten interviews as evidenced by a lack of additional data during the final two interviews.

### Reflexivity and trustworthiness

This study formed part of my (CG) postgraduate studies in Emergency Medicine. During this study I was employed as a lecturer at an SA EMS college. My previous experience with qualitative interviewing was gathering EMS provider perspectives on palliative care. My personal bias is in favour of EMS and palliative care integration. However, several participants raised valid concerns which must be considered. The framework of Guba was applied to ensure data trustworthiness regarding credibility, transferability, dependability and confirmability [24]. Table 1 denotes the methods by which these criteria were pursued.

**Table 1:** Methods employed to ensure data trustworthiness.

**Credibility**	Pilot interview, opportunity for participants to refuse participation, frequent debriefing sessions among research team, researcher triangulation, member checking (resulting in no changes), reflexive commentary.
**Transferability**	Thorough description of data collection methods including number and length of interviews and the data-collection time-period, results compared with available literature, use of the *COnsolidated Criteria for Reporting Qualitative* (COREQ) research checklist [25].
**Dependability**	Thorough descriptions of research design, implementation, and data collection, reflexive commentary.
**Confirmability**	Researcher triangulation, member checking, *verbatim* transcriptions of audio-recordings produced.

Ethical approval for the study was provided by the UCT Human Research Ethics Committee (HREC Reference Number: 220/2023).

## Results

Ten participants were interviewed (Table 2). Interviews lasted between 34 and 57 minutes. Four categories, with subcategories, were developed (Table 3). Participants maintained an overall positive view of EMS and palliative care integration, noting the beneficial impact of EMS and suggesting various methods of integration, while also highlighting challenges and concerns.

**Table 2:** Interviewee and interviewer details.

	Gender	Qualifications	Post-Grad Palliative Experience	Environment
**Interviewee #1**	Female	Medical Doctor - specialised in family medicine, PGDip Palliative Medicine, MSc Palliative Medicine, PhD Palliative Medicine	30 years	Urban
**Interviewee #2**	Female	Medical Doctor - specialised in family medicine, MPhil Palliative Medicine, PGDip Health Professions Education	19 years	Urban
**Interviewee #3**	Male	Medical Doctor, Palliative Care Short Course	9 years	Urban and Peri-Urban
**Interviewee #4**	Female	Medical Doctor, MPhil Palliative Medicine	27 years	Urban
**Interviewee #5**	Female	Professional Nurse, Short Course Palliative Care	9 years	Urban
**Interviewee #6**	Female	Medical Doctor, Psychiatrist, Master's in Early Childhood Intervention, PGDip Interdisciplinary Pain Management, PGDip Palliative Medicine	4 years	Peri-Urban and Rural
**Interviewee #7**	Female	Medical Doctor, PGDip HIV Management, MSc Palliative Medicine	8 years	Urban
**Interviewee #8**	Female	Professional Nurse, Bachelor of Social Science, PGDip Palliative Medicine	7 years	Urban
**Interviewee #9**	Female	Medical Doctor, PGDip Palliative Medicine, Mphil Palliative Medicine, Mphil Bioethics	9 years	Urban
**Interviewee #10**	Female	Intermediate Life Support^1^, Medical Doctor - specialised in emergency medicine, PGDip Palliative Medicine	5 years	Urban and Peri-Urban
**Interviewer (CG)**	Male	Emergency Care Practitioner^2^, MPhil Emergency Medicine, Palliative Care Short Course^3^	10 years (EMS experience)	Urban and Peri-Urban

PGDip = Postgraduate Diploma, MSc = Master of Sciences, PhD = Doctor of Philosophy, HIV = Human Immunodeficiency Virus

1 Vocational paramedic training. Historically this training involved a three-tier system: basic, intermediate, advanced.

2 In SA, an Emergency Care Practitioner is a paramedic with a 4-year Bachelor's Degree in Emergency Medical Care.

3 10-week course through UCT introducing basic palliative care management principles.

**Table 3:** Categories and subcategories developed from interviews.

(1) Disposition Towards EMS	(2) Perceived EMS Challenges	(3) Positive EMS Impact Across Patients’ Palliative Care Journeys	(4) Methods of EMS and Palliative Care System Integration
(a) Calming effect (b) Capabilities in palliative situations (c) Unreliability in service delivery	(a) Persisting EMS and palliative care cultures (b) Decision-making in challenging circumstances (c) Moral distress	(a) Unique insight into determinants of patient health (b) Providing initial containment (c) Liaising with palliative care providers (d) Safe patient transitioning (e) Determining trajectory of care	(a) Collaborative approaches (b) Education of all role-players (c) Potential, unintended consequences

## Discussion

Participants were asked to share their perceptions regarding EMS in palliative situations regarding feasibility, utility and concerns within the SA context. Below, each category is discussed with supporting quotations extracted from interviews. Interviewees are identified by number. (e.g. Interview #1)

### Disposition towards EMS

Overall, participants expressed a positive disposition towards EMS in palliative situations, however, some concerns and negative views were raised. Their disposition was grounded in personal experience and discussed in terms of EMS (a) calming effect, (b) capablities in palliative situations and (c) unreliability in service delivery.

(a) *Calming effect*

Participants noted the calming effect which EMS bring to palliative situations due to their expertise and professionalism, expressing appreciation and respect for the role they play.

*“It may be a small [EMS] role…but I think it's really an important role bringing the patient to us in a comfortable, calm, professional way. I appreciate that.”* – Interview #4

*“…it was a…really hectic case…and it was such a relief to just know that they [EMS] were there.”* – Interview #8

Previous qualitative work in HICs has likewise demonstrated a positive disposition of palliative care providers towards EMS and the reassurance which EMS bring [10,11] Juhrmann et al. [11] noted that EMS held a revered public identity and were perceived as crisis resolvers. An earlier Canadian study found that patients and families with palliative needs experienced peace of mind knowing EMS were available 24/7 [10]. Moreover, participants highlighted the reassurance EMS bring to palliative care providers themselves, particularly in emergency situations. These perceptions align with those of SA EMS providers who likewise perceived palliative care positively [13].

(a) *Capabilities in palliative situations*

While integration was recommended, there were mixed perceptions concerning current EMS capabilities in palliative situations. Some participants expressed confidence in EMS while others expressed negative views.

*“We've used EMS around here and yeah, I'm pretty confident in their skills and expertise, I wouldn't hesitate to call them.”* – Interview #5

*“I think from the doctors’ side of things, there's a lack of respect for for paramedics.”* – Interview #9

Confidence in EMS was based primarily on their clinical abilities in emergency situations. Such expertise is valuable when managing patients with palliative needs as they are prone to abrupt deterioration and acute exacerbations of illness [[26], [27], [28]]. While EMS are trained in the management of some common palliative emergencies, such as seizures, they are untrained in others, for example, superior vena cava obstruction. Thus, several participants questioned EMS provider ability to manage these situations appropriately while others described negative views of EMS providers, perceiving a lack of capabilities and interest, particularly among basic EMS providers.

*“A lot of our…lower tier EMS professionals, just don't have the ability. I mean most of the guys we see… will see a patient [in respiratory distress]…and not do anything about it…So like to try and train those guys up when they're not interested…”* – Interview #7

As basic EMS providers in SA form the majority of the workforce, such perceptions evoke concern. The potential integration of EMS and palliative care in SA across specific EMS qualifications remains an area for study, however, it appears logical that such integration must incorporate basic providers to be of significant benefit. Lamba et al., [29] previously suggested only those EMS providers with a desire for palliative care would be suitable for integration. This may be a beneficial criterion for basic EMS providers in SA to be involved with palliative care.

Several participants further expressed negative views of EMS concerning unnecessary transport. While transport is an important EMS function, it is not always appropriate in palliative situations. Many patients wish to be treated without conveyance to a medical facility [30,31]. EMS were described as *“glorified bus drivers”, “taxi”* and *“transport services”* with participants recounting invariable patient transportation regardless of context. Thus, one participant has suggested patients *“…don't phone an ambulance”* (Interview #8).

These perceptions appear aligned with a large study in the Western Cape province of SA which found that 98% (n=240 730) of patients responded to by EMS were transported to hospital despite 83% (n=199 062) of these not having received any EMS treatment [32]. Within LMICs EMS are frequently used solely for transportation purposes [33] Should EMS and palliative care integration take place in SA, mechanisms for homecare without conveyance, for example a *“treat and release”* approach [5] that is underpinned by an appropriate legislative framework, require development.

(a) *Unreliability in service delivery*
  *“When you call EMS, you are just trying them…to see if they will come or not.” -* Interview #3

*“I would say out in [rural area]…people probably choose a taxi rather than going with ambulance, just cause the taxi is faster than waiting for an ambulance to arrive.”* – Interview #6

Participants distinguished between private and state EMS services within SA. Whereas private EMS were described as reliable, quick and available, state EMS were described as unreliable and problematic. Furthermore, according to participants, while private EMS were willing to transport patients to private hospices, state EMS were unwilling and did not prioritise end-of-life situations resulting in prolonged waiting times for an ambulance.

Due to financial constraints and a lack of transportation options, patients with palliative needs in rural areas are largely reliant on state EMS along with the vast majority of SA citizens who do not hold health insurance (83%) [34]. Compounding the problem is the relative lack of available palliative services in these areas. Within HICs, EMS integration with palliative care has been recommended specifically in rural areas where 24/7 palliative care services are unavailable as EMS may fill this gap [35]. However, within LMICs, this may be of only theoretical benefit as EMS systems are frequently underdeveloped [33]. Despite SA maintaining one of the most developed EMS systems within Africa, [36] the unreliability of (state) EMS, remains a challenge. This unreliability may be due to geographic challenges, the significant number of rural areas, and a lack of rural infrastructure all of which restrict EMS access. In addition, as one participant noted, some areas may be unsafe for both EMS and palliative care providers to enter. Attacks on EMS providers have been frequently reported in SA, resulting in certain areas requiring a police escort [37,38]. For meaningful EMS and palliative care integration in SA, EMS systems should be further capacitated, particularly in rural areas.

### Perceived EMS challenges

While expressing some concerns, participants also perceived challenges faced by EMS providers in palliative situations, potentially inhibiting their abilities to appropriately manage such situations. These challenges were expressed as a) persisting EMS and palliative care cultures, b) decision-making in challenging circumstances and c) moral distress.

(a) *Persisting EMS and palliative care cultures*
  *“That's kind of the traditional…EMS* [life-saving approach]*, but that's not necessarily appropriate in palliative care. It might not be within the patient's goals of care to bring them to hospital.”* – Interview #7

EMS culture is interventionist in nature, through curative approaches in emergency settings, with the aim of saving life and limb [39,40]. Supportive palliative approaches, such as allowing natural death, are relatively foreign to EMS culture. Thus, EMS feel uneasy in palliative situations, as SA EMS providers have detailed [13]. This apparent cultural conflict has been a frequently cited reason for EMS and palliative care segregation [28,40,41]. However, with correct training, EMS and palliative care cultures may be complementary [26,28].

While EMS culture requires a change in palliative situations, participants identified that palliative culture too, may require a shift to allow EMS inclusion. For example, one participant noted palliative care providers often feel protective of their patients and may avoid including other providers. EMS may be particularly avoided as they may provide inappropriate care and transport. Another participant, however, noted that palliative care may benefit from the more structured approach used by EMS and may, therefore, benefit from training concerning EMS.

(a) *Decision-making in challenging circumstances*

Decision-making for EMS in palliative situations was described as difficult due to competing wishes of those involved, minimal system and legal support, and a lack of on-scene information.

*“I also think one of the biggest barriers* [for EMS] *is the medico-legal things that are not unpacked yet and that decision making..”* – Interview #2

*“So there is that very difficult information gap…because there's a mismatch between what the patient and family expect...So I think it is very, very challenging for EMS personnel.”* – Interview #1

Participants noted the unfair nature of expectation upon EMS providers to make challenging decisions under time pressure in isolation. They also detailed the consequences of poor decisions including overlooking patients with unidentified palliative needs and failing to diagnose emergencies in palliative situations which require active intervention. *“Missing”* these emergencies was identified as worse than unnecessary conveyance by EMS. One participant described such a situation in which an EMS provider decided against conveyance due to the palliative nature of the situation when, in fact, conveyance was necessary for emergency treatment. Such difficulties may partly explain why conveyance is the default EMS decision. Murphy-Jones and Timmons [42] remarked that conveyance provides a *“safety net”* for EMS providers, ostensibly providing medico-legal protection. SA EMS providers have likewise identified decision-making challenges in palliative situations for similar reasons, feeling constrained in their approach to palliative situations as a result [13]. To alleviate these challenges, one participant described the potential creation of a palliative care card.

*“You've got a Road to Health for children. Should there be a palliative care card for people who have got a progressive illness…which immediately gives all the clinical information that the EMS personnel needs at that particular time?”* – Interview #1

The Road to Health booklet, in widespread use across SA, keeps a record of children's growth, immunisations, and healthcare interventions from birth [43]. SA EMS providers refer to these booklets when managing paediatrics and are trained in their use. Implementing a similar palliative care booklet represents a potentially low-cost, high-impact intervention which may be of use not only to EMS, but to the broader healthcare system. We highly recommend this intervention be explored.

(a) *Moral distress*
  *“I think we are absolutely not using our resources correctly…if we don't teach our EMS healthcare professionals to provide palliative care. I think we are firstly creating moral distress in our healthcare professionals and therefore they won't stay in the profession long.”* – Interview #2

*“The training of EMS is very algorithmic…[But] sometimes… palliative care, it's not algorithmic.”* – Interview #9

EMS providers globally, including in SA, have expressed their experiences of distress when disregarding their training in palliative situations and have likewise identified the need for palliative care education [13,27,40]. This suggests EMS providers realise an alternate approach is required in palliative situations, however, they are unaware of appropriate strategies. As stated by participants, the ability to effectively calm stressful palliative situations through symptom control, communication strategies, and care planning, relieves moral distress in healthcare workers, however, this cannot be achieved without education. Participants referred to this ability as *“containment”* of palliative situations. Furthermore, they noted healthcare providers themselves require containment to avoid moral distress and, if not adequately equipped, will *“crumble”* in palliative situations. Not only would palliative care integration assist EMS providers in palliative situations, but the containment strategies learned may benefit them more generally as they are often required to contain chaotic emergency situations.

### Positive EMS impact across patients’ palliative care journeys

Despite these challenges and concerns, participants identified the unique position of EMS as intermediaries and its potential positive impact across patients’ palliative care journeys. As EMS arrive in palliative situations they gain a) unique insight into determinants of patient health, b) provide initial containment, c) liaise with palliative care providers, d) safely transition patients, and e) determine trajectory of care.

(a) *Unique insight into determinants of patient health*
  *“…*[EMS] *provide such a unique insight into people's lives…You see people at their worst. So its a great opportunity to network and collaborate.”* – Interview #6

As first-responders, EMS providers frequently enter patient homes. This privileged position within healthcare provides access to valuable information, such as living conditions, socioeconomic status, educational needs, and barriers to care [44]. This unique insight into social and structural determinants of health allows EMS to contribute toward bespoke care plans based on observed circumstances and link patients with relevant services within the broader palliative care team [44]. To leverage this unique insight for palliative care provision may require a mindset shift for EMS providers. Information regarding determinants of health may frequently be overlooked, particularly in emergency situations where their focus is, appropriately, on intervening and transporting. Thus, a need exists for EMS to identify divergent contexts and shift priorities accordingly.

(a) *Providing initial containment*
  *“…*[EMS] *would be like a first-responder…If they could feedback to the home care doctor…could give morphine…and hand over to a homecare team instead of taking the patient to hospital, then that would be really helpful...”* – Interview #8

Initial containment functions would be valuable for palliative care providers as EMS may arrive at palliative situations sooner than home palliative services and relay necessary information. This EMS ability to rapidly respond to an incident and stabilize palliative emergencies before linking the patient with further care would improve patient wellbeing. To achieve participants’ visions of initial containment, avoiding unnecessary conveyance, and homecare team handover, SA EMS providers must be capacitated to do so. This may involve the development of new policies allowing on-scene discharges by advanced EMS providers; a skill within their scope of practice, though not currently used.

(a) *Liaising with palliative care providers*

Participants noted EMS providers may act as their *“eyes and ears”* in palliative situations not only by relaying information, but also implementing management strategies.

*“*[EMS] *need to be able to link with services. You've been called, there's a crisis, an initial assessment of the patient, an understanding that this is…a known palliative care patient…and linking…being able to communicate with the patient's treating providers.”* – Interview #10

Dent et al. [45] demonstrated that palliative telephonic advice provided to EMS resulted in decreased rates of unnecessary transport and improved patient care. Cultivating communication between EMS and palliative care systems represents another potentially low-cost, high-impact intervention. Through increased homecare, the use of EMS as liaisons may decrease overall healthcare costs by reducing unnecessary hospital admissions. Though a cost-benefit analysis is yet to be performed in SA, this benefit has been frequently emphasized in the literature and should be studied as it is particularly relevant to LMIC settings [4,9,35].

(a) *Safe patient transitioning*

Furthermore, participants expounded upon the benefits of safe patient handling and transportation by EMS. EMS provider handling of patients with palliative needs was described as pleasant and professional.

*“For a patient to get into a family's car, it's horrific. When you can't walk properly and you drag them out and you're pulling and pushing them and they're ill, they can't sit up…but somehow* [with EMS] *it just seems more professional. It seems more controlled.”.* – Interview #4

While EMS transport may be beneficial, two participants questioned whether it necessitated EMS. One recommended dedicated palliative care transport services apart from EMS, while another objected to this, and another enquired whether patient handling and transport could be performed by non-EMS personnel. Within the resource-limited SA setting it has been argued that integrating current EMS and palliative care systems may be more cost-effective than establishing a new specialised unit [12]. Despite differing ideas, participants agreed that safe patient transitioning is an important aspect of palliative situations which EMS are capable of managing.

(a) *Determining trajectory of care*

According to participants, EMS decision-making determines patient trajectory and consequences of inappropriate decisions include artificial life-prolongation, increased healthcare costs, and negative patient and family experience. However, with appropriate training, participants perceived beneficial trajectories being set by EMS.

*“*[EMS] *intubating the patient at the end of life…now the patient lands up in ICU* [Intensive Care Unit]*. It's expensive. There's the trauma of it. The patient is not where they want to be when they die. The family is separated from their loved one at the end of their life. Apart from the pain and discomfort to the actual patient”.* – Interview #7

Breyre et al. [46] found that implementation of an EMS and hospice collaboration, which involved training EMS providers in palliative care, decreased EMS transport rates in palliative situations from 80.3% to 19.6%, allowing more beneficial care trajectories. Given the substantial impact of EMS decision-making in palliative situations on patients, families, healthcare providers and economy, EMS integration with palliative care appears desirable. This may be particularly relevant in LMIC contexts which contain the vast majority of patients with palliative needs globally [47,48] and require efficient use of limited resources.

### Methods of EMS and palliative care system integration

All participants agreed integration was necessary and identified various integrative methods for implementation in SA. These methods were discussed in terms of (a) collaborative approaches, (b) education of all role-players and (c) potential, unintended consequences.

(a) *Collaborative approaches*
  *“So it's about creating awareness in government and policymakers about why palliative care is so important in South Africa…that's where I would start: creating awareness with the public and from the top”.* – Interview #6

*“Research is a very strong integration tool… So I do think…a next step is to do a pilot study”.* – Interview #2

When considering EMS, participants recognized the need for palliative care provider input which is currently lacking. As one participant said, *“You give us input. How much input do we give?”* (Interview #4) To foster this input, areas for collaboration, such as regular, joint EMS and palliative care meetings, were suggested.

In 1990 the Commission on Health Research and Development stated that improving LMIC research capacity is *‘one of the most powerful, cost-effective and sustainable means of advancing health and development’* [49]. Pilot studies within the LMIC context of SA may be of worldwide benefit and should be conducted. We recommend these studies analyse modes of EMS palliative care delivery such as community paramedic models, methods of enhanced EMS and palliative system communication,and the effect of these upon hospital conveyance rates, overall EMS caseload, patient and family member satisfaction. Secondary analyses may include safety and efficacy of *“treat and release”* approaches and expanded EMS scope of practice to include commonly used medications within palliative care.

(a) *Education of all role-players*

Education for all role-players, including EMS and palliative care providers, patients, families, and general public, was identified by participants as the primary method of EMS and palliative care integration.

*“More needs to be done to bring the two services closer to each other. So this can only be done by…giving more training to…EMS so that [they] can understand how palliative care services work and how these two services can collaborate.”* – Interview #3

*“…I don't know what [EMS] study, but if I look at…what we were taught as doctors [about EMS], I was taught nothing.”* – Interview #6

One participant reasoned that palliative care training would *“make for a better paramedic all round.”* (Interview #5). This is likely true as EMS and palliative care systems, despite differing functions, share several key goals including patient quality of life, symptom management, and relief of suffering. Therefore, just as EMS and palliative care integration may improve palliative care provision, it may likewise improve emergency care provision.

Participants identified the following areas for EMS provider education: basic palliative care principles, communication practices, management of common palliative symptoms and emergencies, ethical issues, end-of-life care and situational recognition (i.e. curative vs support care). As no EMS palliative care curriculum in SA exists, these areas may inform curriculum development. Participants recommended a palliative care module for EMS at undergraduate level and an introductory short course for qualified EMS providers.

(a) *Potential, unintended consequences*

Though EMS and palliative care integration may confer benefits, some participants warned of potential consequencessuch as EMS providers misinterpreting end-of-life and emergency situations.

*“The EMS…may also find itself in a situation where you guys are overwhelmed, but also faced by…new situations…having to make very difficult decisions that you wouldn't normally make.”* – Interview #3

The concern regarding overwhelmed EMS has been raised previously [50]. An expanded palliative care role may strain EMS resources due to amplified caseload and incident times. Current evidence has failed to demonstrate this; though such evidence is limited to HICs [35]. Within LMICs, given their greater burdens of palliative needs, this concern may remain valid. However, EMS and palliative care integration may decrease overall caseload through provision of homecare. In SA, for example, many patients in palliative situations use EMS repeatedly throughout a given year for hospital conveyance [12] and many of these may be unnecessary should adequate homecare be available. Thus, through decreased repeat patient cases, overall EMS caseload may decrease in the long-term. Along with current SA EMS palliative caseload, this represents an area for further inquiry.

Concerns raised by participants over potential consequences require thoughtful consideration. While the integration of EMS and palliative care has been recommended internationally, the possibility of unintended consequences remains. It should be recognized that EMS and palliative care integration, while providing benefits, will likely result in novel challenges. This further justifies pilot studies before large-scale implementation.

*“It's very feasible. We [also] don't really have a choice. Families phone ambulances, ambulances go to houses…It's already happening.”* - Interview #10

*“Why haven't we done it yet?”* - Interview #2

Despite these potential consequences, participants concluded the benefits of EMS and palliative care integration outweigh potential risks and integration is both feasible and necessary in SA. Integration would be mutually beneficial to both EMS and palliative care systems, while concurrently benefiting patient well-being and the broader healthcare system through provision of homecare and reduced rates of unnecessary hospital admission. This represents efficacious and judicious use of limited resources within the LMIC context of SA.

## Limitations

This study must be interpreted considering its inherent limitations. The perspectives of participants may not represent those of other palliative care providers within SA or abroad, limiting transferability. Given the voluntary nature of participation, self-selection bias is likely present. However, participants were broadly distributed across SA and situated in diverse contexts which resulted in a range of perspectives. Furthermore, the transferability of perspectives contained here have been discussed in light of similar studies in various contexts.

## Conclusion

The aim of this study was to gather perspectives of palliative care providers in SA concerning EMS in palliative situations. Four categories were developed from the interviews: (1) Disposition towards EMS, (2) Perceived EMS challenges, (3) Positive EMS impact across patients’ palliative care journeys and (4) Methods of EMS and palliative care system integration. Participants demonstrated an overall positive disposition towards EMS while highlighting various concerns and challenges involving EMS use. Despite these challenges, participants described many benefits of EMS use in palliative situations and recommended integration through various methods such as creating awareness, education and collaborative efforts. Many suggested methods, such as palliative care cards for patients and enhancing EMS and palliative care system communication, represent potentially low-cost, high-impact interventions. Pilot studies investigating these suggestions should be conducted.

## Dissemination of results

Results from this qualitative interview study were shared with The Association of Palliative Care Practitioners of South Africa, Association of Palliative Care Centres, the University of Cape Town Division of Interdisciplinary Palliative Care and Medicine and with various EMS service providers.

## Authors' contribution

Authors contributed as follows to the conception or design of the work; the acquisition, analysis, or interpretation of data for the work; and drafting the work or revising it critically for important intellectual content: CG 70%; WS 20% and LG 10%. All authors approved the version to be published and agreed to be accountable for all aspects of the work.

## Declaration of competing interest

The authors declared no conflicts of interest.

## Appendix Supplementary materials

Image, application 1
