# Supplementary material for: South African palliative care provider perspectives on emergency medical services in palliative situations
Source: Afr J Emerg Med. 2024 Aug 30;14(4):231–9. doi: 10.1016/j.afjem.2024.08.007 (PMC11402548; doi:10.1016/j.afjem.2024.08.007)
Supplement: Supplementary file 1 [file mmc1.docx]

**Supplementary Material 1**

**Discussion Schedule**

NOTES TO INTERVIEWER: Statements in *ITALICS* are instructions to the interviewer. Questions and text to be read out are in **BOLD.** Prompts contained in textboxes may also be read out during the interview to encourage further dialogue or elicit further explanation.

FACILITATION: *Before the interview, refer to this discussion schedule to ensure familiarity with the content. Lead the discussion, take notes and operate the audio recording device.*

PREPARATION: *Audio recording equipment should be tested from various positions within the room before the interview in the case of in person interviews. In the case of virtual interviews, confirm stable internet connectivity and correct operation of the recording function on the chosen platform. Ensure that participant consent forms and note-taking materials are prepared. Ensure the participant is as comfortable as possible before beginning the interview. Confirm that the participant has signed the consent form and that they consent to being audio recorded.*

CONFIDENTIALITY STATEMENT: **There are no right or wrong answers or opinions on the topic we will be discussing. We are here to gather your own personal and valuable perspectives. All of your answers will be kept strictly confidential. I would also like to remind you that your consent may be withdrawn at any time, including during this process.** *(Give opportunity for questions concerning confidentiality)*

SESSION INTRODUCTION: *Briefly introducing yourself. Ask the participant to introduce themselves. You may start the session as follows:*

*Identify participant on the recording by interview number.*

**Thank you very much for taking the time to discuss the topic of palliative care and EMS in South Africa. EMS are typically used for life-threatening, emergency situations with the goal of saving life and limb, primarily in the out of hospital setting (for example in patient homes). In this setting, EMS providers often encounter palliative patients in emergent and non-emergent situations requiring care. Thus, EMS and palliative care overlap in these situations. The purpose of this interview is to gather your perspectives on EMS use in palliative care in terms of its utility, barriers and concerns with use and feasibility in the South African setting.**

**I will begin by briefly asking about yourself and your experience in South African palliative care. I will then ask six main questions on the topic which we can discuss. If, at any point, you have any questions or need clarification please feel free to ask whenever you would like.** *Re-iterate during this introduction that this process is less of a formal interview and more of a discussion to gain the interviewees valuable and informed perspectives. In addition, mention that notes will be taken during the interview, but this is nothing to worry about.*

1. **Could you please begin by telling me about your career and background?**

*PROMPTS AND PROBES:*

- *Demographics: Age, Gender, Qualification, Current Position, Area of Work (rural vs. urban).*

- Years of experience.

- Previous experience/positions.

2. **In a previous study we gathered data on the intersection between EMS and patients with palliative needs. With regards to your setting, how do these findings compare?**

*PROMPTS AND PROBES:*

- *In a state district hospital, 51% of patients with palliative needs used EMS, and 27% in a state tertiary hospital.*

- *This resulted in up to 36 such EMS palliative cases per month at a single hospital.*

- *The vast majority of patients with palliative needs (84%) were from rural areas and were cared for primarily by family (89%).*

- *The leading chief complaint was dyspnoea (36%) while the leading diagnosis was cancer (32%).*

3. **What are your thoughts surrounding EMS involvement in palliative situations?**

*PROMPTS AND PROBES:*

- Helpful vs. Harmful.

- Any personal experience with EMS use in palliative care?

- Could EMS potentially be utilised? If so, where?

- *Opportunity for EMS use in palliative care (after hours, palliative emergencies, home care).*

- *EMS perspective from previous study: they often encounter palliative patients, but have limited management options due to system, mindset, resource and education problems. However, they see palliative care as important to their role.*

4. **Would you have any concerns with using EMS in palliative care situations?**

*PROMPTS AND PROBES:*

- Particularly in the out of hospital setting.

- System concerns: lack of integration and communication.

- Legalities.

- Ethical considerations.

- Concerns with EMS: knowledge, education, skill, role/function.

- Concerns for palliative patients: standards of care, inappropriate care

- (Note: answers to this question may naturally flow into the next question).

5. **What barriers exist (if any) in your setting to the potential use of EMS in palliative situations?**

*PROMPTS AND PROBES:*

- Resources

- Communication

- Uncertainty regarding EMS systems and capabilities

- Palliative care system constraints

6. **Taking all of our discussion into account, would you please provide your thoughts on the feasibility of EMS use in palliative situations?**

*PROMPTS AND PROBES:*

- Potential solutions to concerns/barriers.

- *Summary statement: Can/should it be done and how? (or why not?)*

SESSION CONCLUSION: *Once all the questions have been asked and answered you may conclude the session as follows:*

**Thank you so much for spending your valuable time discussing this topic with me and taking part in this study. Your perspectives are incredibly helpful, and I have thoroughly enjoyed our discussion. Your contribution is much appreciated.**

*After the session has been concluded, answer any further questions the interviewee may have concerning the study.*
